# Intracrine Formation of Steroid Hormones in Breast Cancer, Epidermal Keratinocyte, Dermal Fibroblast, and Adipocyte Cell Lines Measured by LC-MS/MS

**DOI:** 10.3390/ijms26031188

**Published:** 2025-01-30

**Authors:** Emre Karakus, Andreas Schmid, Andreas Schäffler, Stefan A. Wudy, Joachim Geyer

**Affiliations:** 1 Institute of Pharmacology and Toxicology, Faculty of Veterinary Medicine, Biomedical Research Center Seltersberg (BFS), Justus Liebig University, 35392 Giessen, Germany; joachim.m.geyer@vetmed.uni-giessen.de; 2 Department of Internal Medicine III, Giessen University Hospital, Justus Liebig University, 35392 Giessen, Germany; andreas.schmid@innere.med.uni-giessen.de (A.S.); andreas.schaeffler@innere.med.uni-giessen.de (A.S.); 3 Steroid Research & Mass Spectrometry Unit, Pediatric Endocrinology and Diabetology, Giessen University Children’s Hospital, Justus Liebig University, 35392 Giessen, Germany; stefan.wudy@paediat.med.uni-giessen.de

**Keywords:** intracrine signaling, sex steroids, MCF-7, HaCaT, human dermal fibroblast, 3T3-L1 adipocytes, LC-MS/MS

## Abstract

Peripheral tissues such as skin and adipose tissue play a crucial role in the intracrine formation of sex steroid hormones, complementing the endocrine and paracrine systems. These mechanisms involve the conversion of dehydroepiandrosterone (DHEA) and its sulfated form—DHEAS—into potent androgenic and estrogenic hormones. In vitro studies using tissue-specific cell lines are essential for unraveling the complex intracrine synthesis of these hormones. This study examined the formation of DHEA, androstenedione (A4), testosterone (T), dihydrotestosterone (DHT), and estradiol (E2) from DHEAS in four cell lines: MCF-7 breast cancer cells, HaCaT keratinocytes, human dermal fibroblasts (HDF), and 3T3-L1 preadipocytes and mature adipocytes, using liquid chromatography–mass spectrometry (LC-MS/MS). MCF-7 cells converted DHEAS to DHEA, A4, T, E2, and DHT, while HaCaT cells produced all these steroids except DHT. Mature 3T3-L1 adipocytes produced DHEA, A4, T, and DHT. By contrast, HDF and 3T3-L1 preadipocytes converted DHEAS only to DHEA and A4. This study highlights the vital role of peripheral tissues, such as skin and adipose tissue, for the intracrine formation of sex hormones and underlines the crucial role of in vitro cell culture models to analyze such effects. The data shed light on the significant impact of androgen metabolism in skin and adipose tissue, which is of great relevance for aging, wound healing, obesity, and lipid metabolism.

## 1. Introduction

The synthesis, regulation, and signaling actions of sex steroid hormones are fundamental aspects of endocrine physiology. Apart from their synthesis and release from the gonads, androgens and estrogens can be locally formed in peripheral tissues from the precursor molecules dehydroepiandrosterone (DHEA) and its sulfated form—DHEAS [1]. Thereby, DHEA and DHEAS that originate from the adrenals in humans contribute to the overall steroid regulation of peripheral tissues via local steroid production and intracrine signaling [2]. Plasma levels of DHEA and DHEAS decrease in both men and women with age [3,4], and this decrease has been associated with various age-related conditions, including cardiovascular disease [5], prostatic [6] and breast cancer [7,8], immune deficiency [9], poor mental health [10,11], and rheumatoid arthritis [12], highlighting the physiological importance of these steroid precursors.

The skin is recognized as the largest organ of the body and a major site of intracrine sex steroid hormone production. In addition, the skin is a dominant target organ for steroid regulation [13]. It is well known that estrogens significantly influence skin physiology and aging. Postmenopausal women with estrogen insufficiency experience increased oxidative stress in the skin, atrophic skin changes, accelerated skin aging, and impaired wound healing [14,15,16]. Although the intracrine production of sex steroids and their regulatory function via steroid receptors play a crucial role in skin physiology, the complex pathways involved are still poorly understood. Similarly, adipose tissue emerges as a significant site for intracrine sex steroid hormone synthesis, where DHEAS acts as a pivotal reservoir to produce active androgens and estrogens [17]. Decreasing plasma levels of DHEA and DHEAS have been associated with obesity-related parameters, such as a high body mass index [18,19], central fat accumulation [20], increased visceral fat area in men, and increased waist-to-hip ratio in women [21].

In vitro studies using tissue-specific cell lines are important to understand the complex mechanisms of intracrine sex hormone formation and signaling [22]. Cell line models offer researchers reproducible and scalable platforms to investigate complex metabolic pathways, dose–response relationships, and temporal dynamics of intracrine sex steroid production and action [23]. In addition, cell line models are valuable tools for the screening of potential therapeutic agents targeting intracrine pathways. They provide promising avenues for the development of novel treatment strategies for diseases such as cancer, metabolic disorders, and inflammatory conditions [24]. In vitro studies in cell line models are also essential to advance our understanding of intracrine signaling and its impact on cellular balance and disease development [13].

We already demonstrated some years ago that a large spectrum of sulfated steroids can be detected in cell lysates from HEK293 cells overexpressing the steroid sulfate uptake carrier NTCP using liquid chromatography–mass spectrometry (LC-MS/MS) methodology [25]. The aim of the present study was to investigate the intracrine androgen and estrogen formation from DHEAS as the precursor in different tissue-specific cell lines that were not transfected with any steroid sulfate uptake carrier. The cell lines used were MCF-7 breast cancer cells, HaCaT epidermal keratinocytes, human dermal fibroblasts (HDF), and 3T3-L1 preadipocytes and mature adipocytes. Among them, MCF7 and HaCaT converted DHEAS to testosterone (T) and estradiol (E2), while mature 3T3-L1 adipocytes only produced dihydrotestosterone (DHT) and T, not E2, after incubation with DHEAS.

## 2. Results

### 2.1. Analysis and Quantification of Steroid Hormones by LC-MS/MS

**Figure 1:**
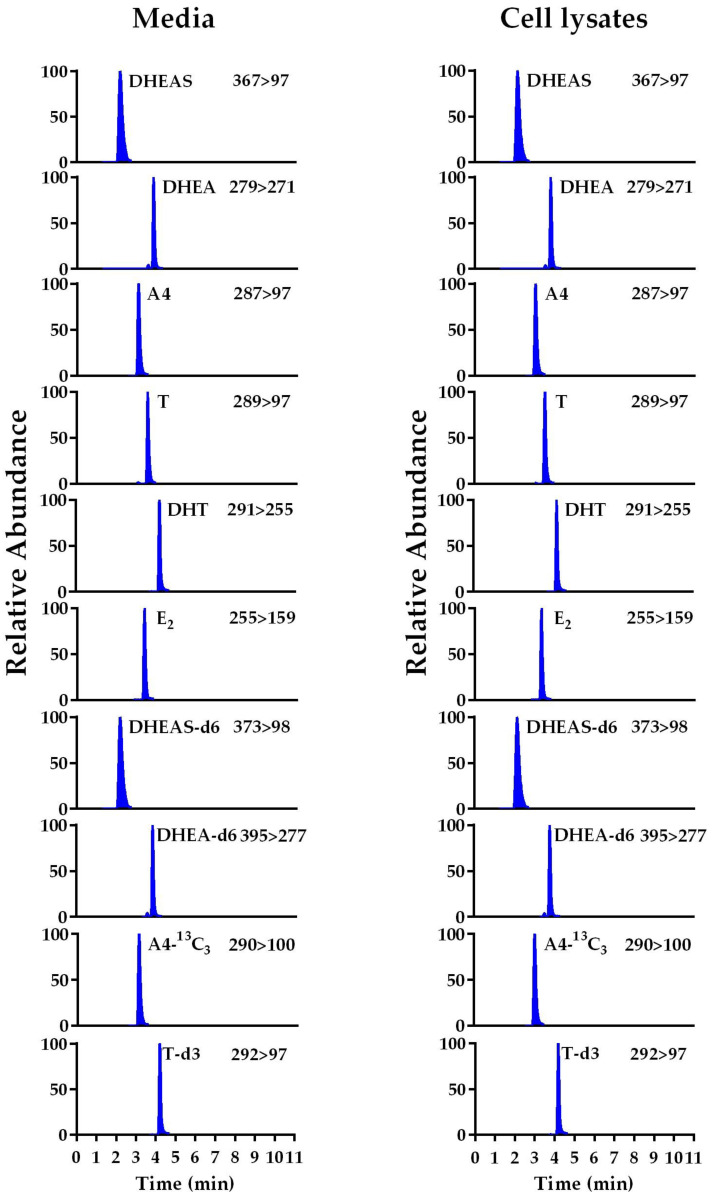
Representative MRM chromatogram of LC-MS/MS analysis of cell culture medium and cell lysate. The samples were spiked with 500 ng/mL of each analyte. The *m*/*z* values (mass-to-charge ratios) are indicated.

Direct infusion was used to determine the optimal mass spectrometry conditions for analysis and quantification of the following steroids: DHEAS, DHEA, androstenedione (A4), T, DHT, and E2. The spectra for the analytes DHEAS, DHEA, A4, T, DHT, and E2, and the stable-isotope-labeled internal standards (ISs) DHEAS-d6, DHEA-d6, A4-^13^C_3_, and T-d3 showed protonated molecular ions at *m*/*z* of 367.2, 289.1, 287.2, 289.1, 291.1, 255.2, 373.2, 295.1, 290.2, and 292.1, respectively. Their collision-induced dissociation formed a distinctive product at m/z values of 96.9, 271.3, 97.1, 97.0, 255.3, 159.0, 97.7, 277.1, 100.1, and 97.0, respectively (Table 1 and Supplementary Figure S1). The multiple reaction monitoring was based on the following transitions: *m*/*z* of 367.2 > 96.9, 289.1 > 271.3, 287.2 > 97.1, 289.1 > 97.0, 291.1 > 255.3, 255. 2 > 159.0, 373.2 > 97.7, 295.1 > 277.1, 290.2 > 100.1, and 292.1 > 97.0 for DHEAS, DHEA, A4, T, DHT, E2, DHEAS-d6, DHEA-d6, A4-^13^C_3_, and T-d3, respectively. The retention times (RTs) for the steroids and their corresponding IS values were 1.85 min for DHEAS, 3.91 min for DHEA, 3.21 min for A4, 3.65 min for T, 4.28 min for DHT, 3.42 min for E2, 1.82 min for DHEAS-d6, 3.87 min for DHEA-d6, 3.20 min for A4-^13^C_3_, and 3.61 min for T-d3, respectively (Table 1 and Figure 1). The individual samples were each run for a total of 10 min in the chromatographic system (Figure 1).

**Figure 2:**
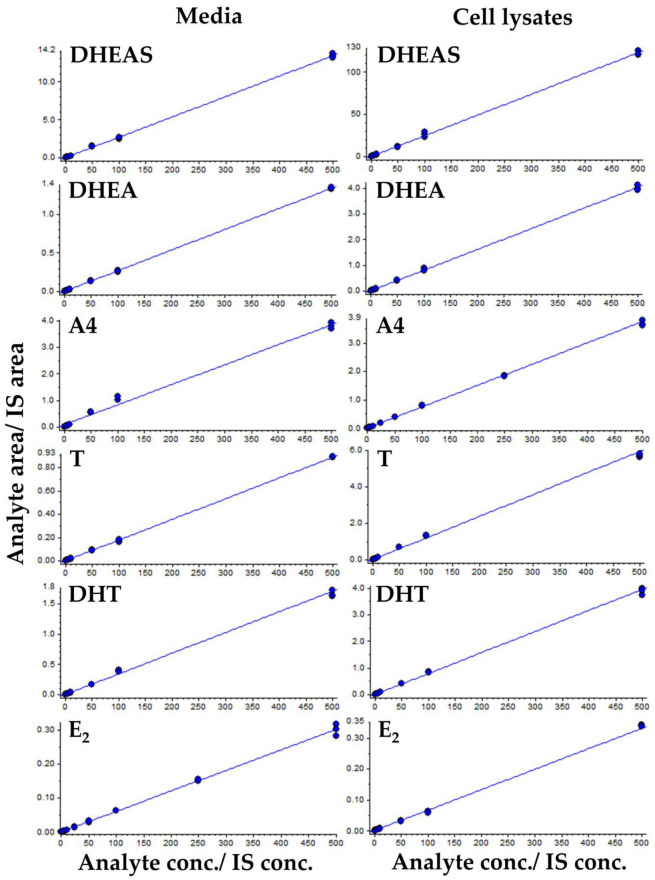
Representative calibration curves of the method for each steroid analyte in media and cell lysates. The peak area ratio of the respective analyte and IS are plotted against the concentration of the analyte; linear fit and 1/x weighting are used.

Calibration samples were prepared at seven different concentrations from 0.5 to 500 ng/mL for each analyte. Linear regressions with 1/x weighting values were used to generate calibration curves for DHEAS, DHEA, A4, T, DHT, and E2. The coefficient of determination (R^2^) values for these curves in the cell culture media were 0.998, 0.999, 0.998, 0.997, 0.998, and 0.997, respectively. Similarly, the R^2^ values for the corresponding analytes in cell lysates were determined to be 0.998, 0.999, 0.997, 0.998, 0.999, and 0.997, respectively (Table 2 and Figure 2). The lower limit of quantification (LLOQ), characterized as the minimum concentration at which both precision and accuracy were below 20%, for DHEAS, DHEA, A4, T, DHT, and E2 in cell media was determined to be 0.994, 5.521, 0.538, 2.372, 2.201, and 5.1 ng/mL, respectively. The LLOQ values for DHEAS, DHEA, A4, T, DHT, and E2 in the cell lysates were 0.957, 4.938, 0.482, 2.385, 2.575, and 5.059 ng/mL, respectively (Table 2).

The reproducibility of the method was assessed through intra- and inter-assay precision and accuracy evaluations for the LLOQ and quality control (QC) samples at low (LQC), medium (MQC), or high (HQC) concentrations. These assessments are summarized in Supplementary Table S1 for the cell culture media and in Supplementary Table S2 for the cell lysates. The results confirm the reproducibility and repeatability of the analytical process.

The recovery after solid-phase extraction (SPE) ranged from 93.5 (A4) to 120.4% (DHEAS) for LQC, from 90.5 (DHEA) to 115.2% (T) for MQC, and from 89.0 (DHEA) to 108.8% (A4) for HQC in the cell culture media. Recovery from cell lysates ranged from 92.3 (DHEAS) to 110.0% (DHEA) for LQC, from 88.4 (DHEA) to 115.2% (E2) for MQC, and from 91.2 (DHEA) to 117.0% (E2) for HQC. Almost all analytes exhibited low relative error across different concentration levels, indicating consistent purification. The analysis of steroids in both cell culture media and cell lysates revealed a negligible matrix effect, as shown in Supplementary Table S3. The peak areas obtained from injecting standard solutions in the mobile phase were compared with those of standard solutions added to extracted sample solutions from cell culture media and cell lysates, resulting in values ranging from 87.7 to 113.8% in the media and from 83.2 to 110.3% in the cell lysates.

The stability tests indicated that there was no significant degradation of steroids during four freezing and thawing cycles over periods of 6, 24, and 56 h at room temperature, +4 °C, and –20 °C, as demonstrated in Supplementary Table S4.

### 2.2. Formation of DHEAS Metabolites in MCF-7 Cells

We studied the cellular production of active androgens and estrogens in media and cell lysates from MCF-7, HaCaT, HDF, and 3T3-L1 cells after incubation with 1, 10, or 100 µM DHEAS over 24, 48, or 72 h. The concentrations of DHEAS, DHEA, A4, T, DHT, and E2 were determined by LC-MS/MS in all experiments. The steroid concentrations were generally higher in the cell culture media than in the cell lysates in all cell lines analyzed. It was noteworthy that the concentrations of DHEAS, DHEA, A4, T, DHT, and E2 in the control media without DHEAS addition, as well as in the cell lysates without DHEAS incubation, were all below the detection level.

**Figure 3:**
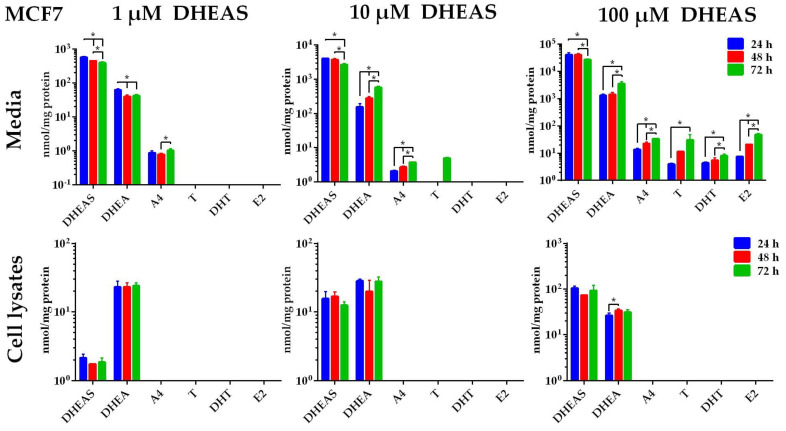
Time- and concentration-dependent metabolism of DHEAS in MCF-7 cells. Cells were incubated with 1, 10, or 100 µM DHEAS. After 24, 48, or 72 h, the media were collected, and the cells were harvested after being washed with phosphate-buffered saline (PBS). The samples underwent analysis for DHEAS and its metabolites using LC-MS/MS. Data represent the means ± SDs of three independent experiments, each with triplicate determinations. * Significant increase or decline between the incubation groups (24, 48, and 72 h) with *p* < 0.05.

MCF-7 cells showed a statistically significant decrease in DHEAS in the cell culture media over time, and a certain amount of DHEAS could be detected from the cell lysates, indicating DHEAS uptake into the cells (Figure 3). In addition, DHEA could be detected in all cell lysates with relatively constant levels over time. The DHEA concentrations were significantly lower in the cell lysates than in the cell culture media. After incubation with 100 µM DHEAS for 72 h, for instance, the concentration of DHEA in the medium reached 3520.2 ± 669.9 nmol/mg protein, whereas only 31.0 ± 4.3 nmol/mg protein of DHEA was detected in the cell lysate (Figure 3). After incubation with 1 µM DHEAS, only DHEAS, DHEA, and A4 were detected in the medium. However, incubation with 100 µM DHEAS resulted in statistically significant increases in the concentrations of DHEA, A4, T, DHT, and E2 at 24, 48, and 72 h (*p* < 0.05), while a statistically significant decrease in DHEAS in the cell culture medium was observed over time. The steroids DHT and E2 were only detectable after incubation with 100 µM DHEAS across all incubation times in the media. The concentrations of DHT and E2 were as follows: 4.3 ± 0.5 and 7.0 ± 0.5 nmol/mg protein at 24 h, 5.5 ± 1.3 and 20.9 ± 0.4 nmol/mg protein at 48 h, and 8.1 ± 1.1 nmol/mg protein and 47.6 ± 6.3 nmol/mg protein at 72 h, respectively (Figure 3).

### 2.3. Formation of DHEAS Metabolites in HaCaT Cells

**Figure 4:**
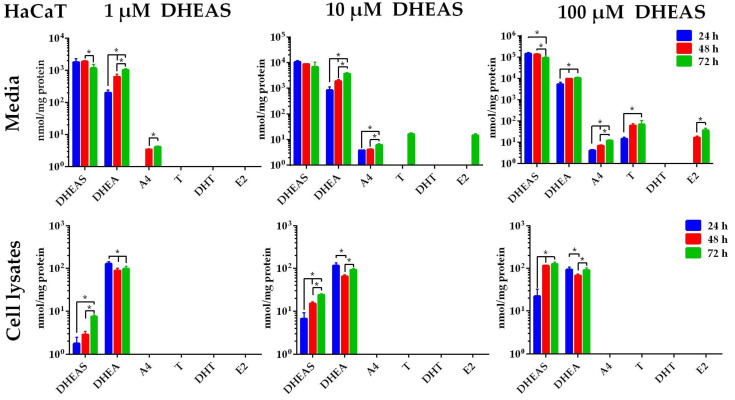
Time- and concentration-dependent metabolism of DHEAS in HaCaT cells. Cells were incubated with 1, 10, or 100 µM DHEAS. After 24, 48, or 72 h, the media were collected, and the cells were harvested after washing with PBS. The samples underwent analysis for DHEAS and its metabolites using LC-MS/MS. Data represent the means ± SDs of three independent experiments, each with triplicate determinations. * Significant increase or decline between the incubation groups (24, 48, and 72 h) with *p* < 0.05.

The results obtained in HaCaT cells (Figure 4) were basically comparable to the findings in MCF-7 cells. The concentration of DHEAS decreased in the media and increased in the cell lysates over time, while the concentration of DHEA in the media increased to levels comparable to those observed in MCF-7 cells.

After 24 h of incubation with 1 µM DHEAS, the concentration of DHEA in the medium was 202.2 ± 42.9 nmol/mg protein and increased significantly to 638.8 ± 131.1 nmol/mg protein after 48 h and to 1035.2 ± 94.2 nmol/mg protein after 72 h of incubation compared to 24 h (*p*  <  0.05). A similar effect was detected following incubation with 10 or 100 µM DHEAS. A4 was detectable after 48 and 72 h of incubation with 1 µM DHEAS (3.4 ± 0.2 and 4.1 ± 0.3 nmol/mg protein, respectively). With 10 µM DHEAS incubation, A4 was detectable at all time points, namely, after 24 (3.8 ± 0.1 nmol/mg protein), 48 (4.1 ± 0.4 nmol/mg protein), and 72 h (6.1 ± 0.0 nmol/mg protein). The steroids T and E2 were also detectable after incubation with 10 µM DHEAS. However, this was only observed after 72 h with concentrations of 16.5 ± 1.6 (T) and 14.7 ± 2.5 nmol/mg protein (E2). The steroid T was detectable in the 100 µM DHEAS incubation group at all three time points (at 14.7 ± 2.5, 62.2 ± 12.3, and 69.7 ± 35.0 nmol/mg protein, respectively). The E2 was detectable after 100 µM DHEAS incubation over 48 and 72 h with levels of 16.7 ± 2.9 and 36.6 ± 8.5 nmol/mg protein, respectively. The steroids T, A4, and E2 were detectable, while DHT remained below the detection limit in all samples (Figure 4).

### 2.4. Formation of DHEAS Metabolites in HDF Cells

**Figure 5:**
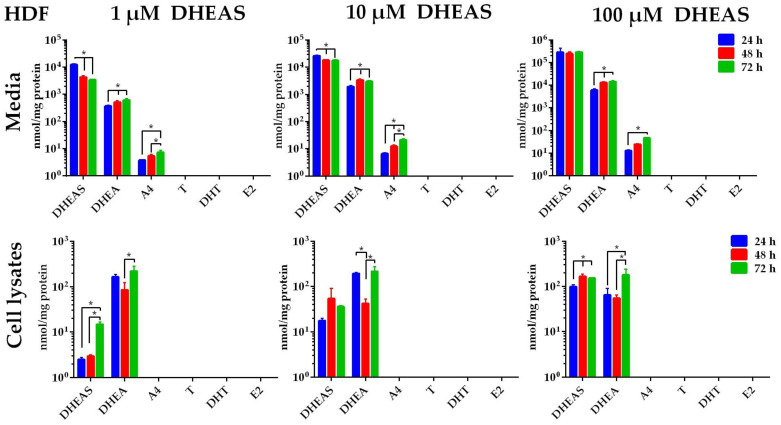
Time- and concentration-dependent metabolism of DHEAS in HDF cells. Cells were incubated with 1, 10, or 100 µM DHEAS. After 24, 48, or 72 h, the media were collected, and the cells were harvested after washing with PBS. The samples underwent analysis for DHEAS and its metabolites using LC-MS/MS. Data represent the means ± SDs of three independent experiments, each with triplicate determinations. * Significant increase or decline between the incubation groups (24, 48, and 72 h) with *p* < 0.05.

In contrast to the MCF-7 and HaCaT cells, the HDF were not able to convert DHEAS to T or E2. As seen for the 1 µM DHEAS incubation medium, the DHEAS concentrations declined, while the DHEAS concentrations increased in the cell lysates (Figure 5). This was accompanied by increasing concentrations of DHEA in the cell culture media over time, starting at 366.8 ± 37.1 nmol/mg protein after 24 h and ending at 617.2 ± 83.8 nmol/mg protein after 72 h of incubation. Similar effects were detected when DHEAS was incubated at concentrations of 10 or 100 µM. The A4 concentrations increased over time in the media but not in the cell lysates, appearing to rise proportionally with the DHEA levels. After 24 h of treatment with 1 µM DHEAS, for example, A4 levels were at 3.8 ± 0.2 nmol/mg protein, increasing to 5.3 ± 0.8 nmol/mg protein after 48 h and 7.3 ± 1.4 nmol/mg protein after 72 h of incubation (Figure 5). On the other hand, levels of T, DHT, and E2 could not be determined in either the cell media or the cell lysates of HDF.

### 2.5. Formation of DHEAS Metabolites in 3T3-L1 Adipocytes

**Figure 6:**
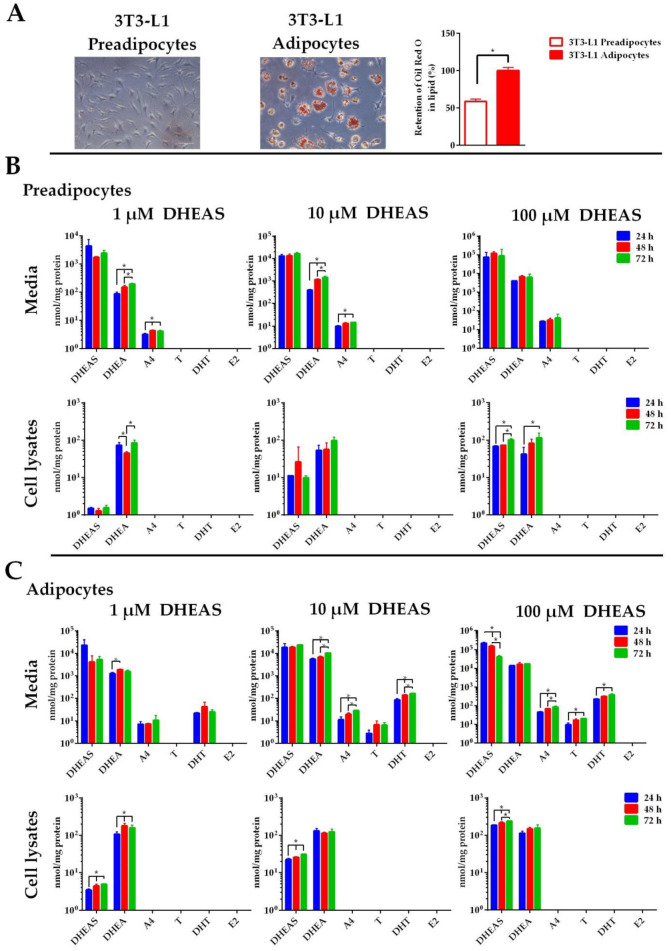
Time- and concentration-dependent metabolism of DHEAS in 3T3-L1 preadipocytes and adipocytes. (**A**) After nine days of adipogenesis in adipogenic differentiation medium, the 3T3-L1 preadipocytes and mature adipocytes were stained with Oil Red O (20 × magnification; scale bar  =  75 μm). Lipid accumulation was quantified by measuring the 510 nm absorbance after eluting the deposited Oil Red O with isopropanol. The undifferentiated preadipocyte cells were directly compared to the differentiated adipocytes. Values for the lipid accumulation are expressed as the means  ±  SDs. Twenty-four hours after seeding (**B**) or after differentiation (**C**), cells were incubated with 1, 10, or 100 µM DHEAS. After 24, 48, or 72 h, the media were collected, and the cells were harvested after washing with PBS. The samples underwent analysis for DHEAS and its metabolites using LC-MS/MS. Data represent the means ± SDs of three independent experiments, each with triplicate determinations. * Significant increase or decline between the incubation groups (24, 48, and 72 h) with *p* < 0.05.

Finally, 3T3-L1 preadipocytes and mature adipocytes were used to study DHEAS metabolism. Accordingly, preadipocyte cells were differentiated into mature adipocyte cells as described in Section 4. After nine days of cultivation in the differentiation medium, lipid droplet formation was assessed and quantified using Oil Red O staining (Figure 6A). Red lipid droplets could be clearly identified under the microscope, and statistical evaluation revealed a significant increase in lipid droplet formation and lipid accumulation in the mature adipocyte cells compared to preadipocytes (Figure 6A). While the preadipocytes converted DHEAS only to A4 (Figure 6B), 3T3-L1 cells started to convert a substantial proportion of DHEAS to T and DHT after differentiation into mature adipocytes (Figure 6C). DHT increased proportionally with DHEA in the cell culture medium at all incubation times and DHEAS concentrations. As an example, DHEA levels increased from 1262.0 ± 185.6 to 1563.2 ± 238.5 nmol/mg protein after 24 and 72 h incubation, respectively, after incubation with 1 µM DHEAS. Concurrently, A4 concentrations showed a similar increasing pattern, starting from 7.0 ± 2.1 nmol/mg protein at 24 h incubation and ending at 10.7 ± 6.4 nmol/mg protein after 72 h of incubation. Finally, DHT increased from 22.0 ± 1.8 to 25.0 ± 6.1 nmol/mg protein, respectively. It is noteworthy that the DHT concentrations exceeded the concentrations of T in the cell culture media at all time points and DHEAS incubation groups. The overall highest concentrations of DHT were detected after incubation with 100 µM over 72 h, reaching 376.4 ± 50.9 nmol/mg protein (Figure 6C).

The steroids DHEA and DHEAS were detectable in preadipocyte cell lysates at all DHEAS concentrations. However, a statistically significant time-dependent increase was only observed at incubations with 100 µM DHEAS (Figure 6B). By contrast, a time-dependent significant increase in DHEAS and DHEA was observed in mature adipocyte cells even at the lowest DHEAS concentration. However, A4, T, DHT, and E2 were not detected in lysates of either preadipocytes or mature adipocytes.

## 3. Discussion

The present study aimed to investigate the intracrine formation of androgens and estrogens using LC-MS/MS methodology in various tissue-specific in vitro cell culture models, including MCF-7, HaCaT, HDF, 3T3-L1 preadipocytes, and mature 3T3-L1 adipocytes. Our findings provide valuable insights into the complex pathways involved in intracrine signaling and shed light on the implications for physiological and pathological processes.

Biologically active steroid hormones are secreted from endocrine organs, such as the adrenal gland, gonads, and placenta, and their synthesis is tightly regulated via the hypothalamic–pituitary–steroidogenic gland axis. These hormones are transported through the circulation within the endocrine system and act via specific receptors at peripheral target cells. In addition to that, bioactive androgens and estrogens in both men and women can be synthesized locally in peripheral target tissues from inactive adrenal precursors, such as DHEA and its sulfate, DHEAS [26]. This local production is commonly referred to as “intracrine” steroid hormone synthesis, and the steroid hormones produced in this way can exert their action in the same (autocrine) or neighboring cells (paracrine) without release into the bloodstream [27]. The intracrine system is considered an efficient mode of hormone action, as it requires minimal amounts of biologically active hormones to exert maximal hormonal effects. This system plays a role in the development of hormone-dependent neoplasms, including prostate, breast, endometrial, and ovarian malignancies. It is crucial to note that serum hormone concentrations may not reflect local hormonal activities in target tissues, emphasizing the importance of studying hormone metabolism within tissue-specific cell lines [28].

**Figure 7:**
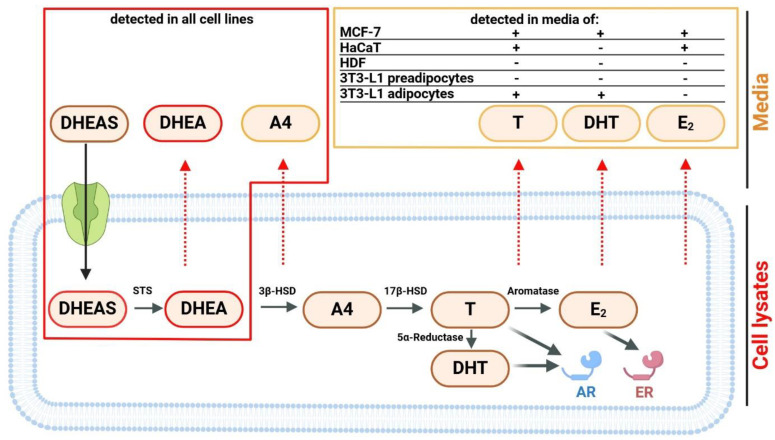
DHEAS metabolic pathway and signaling. Cells were incubated with increasing concentrations of DHEAS and incubated over 24–72 h (see Figure 3, Figure 4, Figure 5 and Figure 6). Steroid concentrations were determined in cell lysates and cell culture media for the DHEAS metabolites indicated. Whereas DHEAS and DHEA were detected in cell lysates and media from all cell lines, T, DHT, and E2 were only detected in the cell culture media of some of the cell lines and, thus, represent a cell-type-specific metabolic phenotype. The steroids T and DHT are potent activators of the AR, while E2 is an agonist of the ER. Black arrow: carrier-mediated uptake of DHEAS; red dotted arrows: passive diffusion of the lipophilic steroids DHEA, A4, T, DHT, and E2. Figure created with BioRender.

Adrenal-derived DHEAS in humans circulates in relatively high plasma concentrations of 1–8 µM in females and 2–10 µM in males and, thus provides a quantitative reservoir for local conversion into bioactive androgens and estrogens in peripheral tissues [29]. Plasma DHEAS levels in adult men and women are nearly 400–4500 times higher than those of T and 100,000–200,000 times higher than those of E2, respectively [29]. DHEAS, as a negatively charged molecule, first needs to undergo carrier-mediated uptake from the bloodstream before it can be desulfated to DHEA by steroid sulfatase (STS) [30]. The DHEA then undergoes further metabolism, catalyzed by 3β-hydroxysteroid dehydrogenase (3β-HSD) to form the intermediate A4 and by 17β-hydroxysteroid dehydrogenase (17β-HSD) to finally form T. The latter can then be further converted into the estrogen E2 by the enzyme aromatase, which is abundant in tissues such as adipose tissue, skin, and ovaries. In addition, T can be converted to the even more potent androgen DHT by a 5α-reductase reaction [17,31,32,33]. The active steroids E2, T, and DHT can then exert their regulatory effects at the estrogen and androgen receptors (ER/AR) that are expressed in the same cells where their intracrine synthesis takes place (Figure 7) [30,33].

Most human breast carcinoma express the ER and are then indicated as hormone- or estrogen-dependent breast carcinoma. In such cases, estrogens such as E2 significantly promote the growth and development of these carcinomas [34]. However, after menopause, estrogens are no longer derived from the ovaries but are primarily produced through local conversion of androgens of adrenal origin, such as DHEAS [35]. Apart from breast carcinoma, this process has also been described for many other peripheral tissues, including the skin [36], fat and muscle [37], and bone [33]. Apart from the ER, the AR is also expressed in breast cancer cells, and DHT has been shown to potently inhibit the E2-mediated stimulatory effect on cell proliferation [38]. The steroid DHEAS stimulated cell growth in the MCF-7 breast cancer cell model, also used in the present study, but reduced the E2-induced cell proliferation, whereas T and DHT inhibited the MCF-7 cell proliferation independent of the presence of E2 [39]. In another study, the MCF-7 cell proliferation was stimulated by single incubations with physiological concentrations of 1 µM DHEAS or 10 nM E2 to the same extent [22]. These data clearly show that the exact composition of the DHEAS-derived steroid metabolome has an impact on the proliferation of these breast cancer cells. MCF-7 cells are one of the most studied human breast cancer cell lines [24] and have been extensively used for in vitro proliferation experiments with different androgens and estrogens [40]. In addition, the steroid metabolic activity of MCF-7 cells has previously been described after incubation with DHEA or estrone. While DHEA was converted to T, estrone was metabolized to E2 [41]. In the present study, we started from the most quantitative precursor DHEAS and, thus, also included the process of the active uptake of DHEAS into the target cells. With this approach, we basically confirmed previous data showing that MCF-7 cells can convert DHEAS to T [41]. In addition, we were able to demonstrate that E2 can be formed not only from estrone but also from high DHEAS concentrations through a process involving active transport and at least four different enzymatic conversions (Figure 7). All of them have already been described to be expressed in MCF-7 cells, namely, STS, 3β-HSD, 17β-HSD, and aromatase [42,43]. However, DHT could not be detected in MCF-7 cells even under 72 h incubation with 100 µM DHEAS in the present study (Figure 3), pointing to a low or absent expression of 5α-reductase.

Estrogens and their receptors also play a direct role in several age-related diseases. As an example, the role of estrogen deficiency in accelerating skin aging is well-documented [14,44,45]. On the other hand, DHEA can effectively ameliorate skin aging [46]. The skin contains a wide range of enzymes capable of metabolizing both topically applied drugs and endogenous substrates. A significant portion of this metabolic activity occurs within the epidermis. Therefore, there is an increasing interest in studying human skin metabolism through steroid profiling. The HaCaT cell line is a spontaneously immortalized human keratinocyte cell line and represents an appropriate in vitro epidermis model [13]. In the present study, we found that HaCaT cells had a similar metabolic phenotype to MCF-7 cells in the sense that they metabolized DHEAS to both T and E2, at least at high DHEAS concentrations. For direct comparison, we used HDF cells, which represent fibroblast cells in the dermis of the skin. These cells converted DHEAS only to A4; no T, DHT, or E2 could be detected, even after a long (72 h) incubation with very high DHEAS concentrations (100 µM). Functional scratch wound healing assays with primary cultures of human skin epidermal keratinocytes and dermal fibroblasts revealed that E2, DHEAS, and DHEA accelerated cell migration of both cell types, clearly pointing to a vital role of these steroids in skin physiology and diseases. As the STS inhibitor STX64 blocked this effect for DHEAS, the essential role of the cellular uptake and desulfation of DHEAS for local steroid supply was underlined [47]. We were able to confirm the formation of DHEA and E2 in the HaCaT cell model in the present study. Thereby, E2 might have been derived via aromatization from T that could also be detected. The expression of 5α-Reductase type 1 mRNA has been demonstrated in human whole skin biopsies and primary cultures of dermal fibroblasts and epidermal keratinocytes, as well as in HaCaT keratinocytes, sebocytes, and melanoma cells. However, corresponding 5α-reductase enzymatic activity has only been confirmed in sebocytes [47,48]. The lack of DHT detection in HaCaT cells in the present study may therefore be due to the limited enzymatic activity of 5α-reductase in these cells. As the scope of this study was limited to the profiling of intracrine steroid synthesis, expression analysis of the relevant enzymes was not performed.

There are significant differences in the volume and distribution of body fat between men and women, which are largely attributable to the disparate metabolic and hormonal characteristics of the sexes. These variations have a marked impact on the distinct health risks associated with obesity in men and women [49]. The lower prevalence of obesity-related metabolic disorders in women under the age of 50 and the increase of these metabolic disorders after menopause indicate that sex steroid hormones play a central role in these responses [50]. The challenges of working with tissues and primary cell cultures are limited access to samples, high inter-individual variability, and developmental changes due to hormonal treatments. Therefore, in the present study, we used the well-characterized murine adipocyte cell line 3T3-L1 [51] to analyze intracrine steroid hormone formation. It was noteworthy that murine 3T3-L1 adipocytes have been shown to express transcripts encoding both the AR and the ER and, therefore, are highly steroid-responsive [52,53,54]. Additionally, Yokokawa et al. [55] found that DHEA suppressed both the proliferation and adipocyte differentiation of 3T3-L1 cells. In addition, DHEA inhibited lipid accumulation in 3T3-L1 adipocytes. Furthermore, Singh et al. [56] demonstrated that T and DHT inhibited lipid accumulation in 3T3-L1 cells in a dose-dependent manner. This inhibitory effect was significantly blocked by the AR antagonist flutamide [56]. We demonstrated in a previous study that DHEAS reduced the lipid accumulation in mature 3T3-L1 adipocytes. Moreover, we observed that both the STS inhibition by STX64 and AR inhibition by flutamide completely abolished the effects of DHEAS, indicating that the conversion of DHEAS to DHEA and to subsequent androgens is involved in this process [57]. Additionally, Dalla Valle et al. [58] observed the mRNA expression, protein expression, and functional activity of STS in human subcutaneous adipose tissue from both men and women. According to Blouin et al. [59], the mRNA expression of STS is strongly induced by differentiation in primary preadipocyte cultures. In addition to the classical steroidogenic tissues (placenta, adrenal cortex, ovary, and testis), 3β-HSD activity is found in several other tissues, including the adipose tissue, endometrium, epididymis, and skin, where it catalyzes the first step in the intracrine conversion of DHEA to A4 [60]. Trilostane (a 3β-HSD inhibitor) blocked the inhibitory effect of DHEA on 3T3-L1 differentiation, indicating the presence of 3β-HSD enzyme activity in metabolizing DHEA and its subsequent influence on adipocyte development [61]. In a study by Marwah et al. [62] the metabolism of DHEA was examined in differentiated 3T3-L1 adipocytes after 48 h of incubation. It was found that low levels of A4 and T were detected in the cell culture media using LC-MS analysis [62]. Furthermore, Corbould et al. [63] found that adipose tissue from the subcutaneous and omental abdominal region in women expressed the mRNAs of three isoforms of 17β-HSD. In a subsequent study, Corbould et al. [64] observed that preadipocytes cultured from abdominal adipose tissue of women with increased type 3 17β-HSD mRNA expression and enzyme activity converted A4 to T. The steroid A4 can be rapidly metabolized to T within this tissue due to the co-expression of 17β-HSD5 in adipose tissue [65]. Therefore, changes in the circulating level of the precursor DHEA, as well as the local expression of 3β-HSD and 17β-HSD5 may directly influence the generation of active androgens in adipose tissue. The specific mRNA level of aromatase increased 17-fold during the adipogenic conversion and remained elevated in fully differentiated adipocytes [66]. We demonstrated in our study that preadipocyte cells metabolized DHEAS only to DHEA and A4, indicating STS and 3β-HSD activities. By contrast, mature adipocyte cells showed concentrations of T and DHT in addition to DHEA and A4, but not E2, suggesting the presence of STS, 3β-HSD, 17β-HSD, and 5α-reductase enzyme activities but no aromatase activity.

In response to the lack of detection of A4, T, DHT, and E2 in the cell lysates of all analyzed cell lines (MCF7, HaCaT, HDF, and 3T3-L1), several factors can be discussed that could explain this observation. (I) Detection limitation: The intracellular levels of these steroids might be below the detection limit of the LC-MS/MS method used. Given the sensitivity and specificity ranges of the equipment, it is possible that the measurements failed to detect very low concentrations, resulting in false negatives. (II) Intracrine steroid dynamics and metabolism: Intracrine pathways often involve rapid conversion of precursors such as DHEAS into active steroids and subsequent metabolism to inactive metabolites. This rapid metabolism can result in minimal accumulation of intermediates, leading to undetectable levels of A4, T, DHT, and E2 within the cells. (III) Steroid secretion: A4, T, DHT, and E2 intracellularly produced via intracrine steroid synthesis could be rapidly secreted to the extracellular medium, where we were able to detect quite high concentrations of these active steroids after DHEAS incubation. (IV) Time point considerations: While we measured the effects of DHEAS at 24, 48, and 72 h, it is possible that the optimal time point for detecting A4, T, DHT, and E2 within the cell lysates was missed.

Although the present study provides important insights into the intracrine formation of sex steroid hormones using in vitro cell culture models, there are several limitations that must be considered. The validation of our findings with additional methods, such as expression analysis of all relevant enzymes involved in the intracrine steroid synthesis, e.g., by PCR or Western blot, or the use of selective transport and enzyme inhibitors could strengthen the biological relevance of our results. Additionally, the use of in vitro cell models may not fully resemble the in vivo situation. Specifically, the use of human and murine cells, cancer-derived MCF-7 breast epithelial cells, and immortalized HaCaT keratinocytes may not fully represent the profiles gener-ated by their respective normal human cell types. For instance, while MCF-7 cells are widely used as a hormone-responsive breast cancer model, they may not accurately reflect the phenotype of normal breast epithelial cells. A similar concern exists regarding the use of immortalized HaCaT keratinocytes, which may not fully reflect the physiology of nor-mal epidermal keratinocytes. Therefore, subsequent studies should aim to validate these findings in animal models or even human clinical samples to ensure the physiological relevance of the observed metabolic patterns.

In conclusion, this study underscores the pivotal role of peripheral tissues, including skin and adipose tissue, in the intricate intracrine mechanisms governing sex steroid formation, which complements the endocrine and paracrine systems. Our study emphasizes the indispensable role of in vitro studies using cell lines in unraveling the intricate mechanisms of intracrine sex hormone signaling and understanding its implications for cellular homeostasis and disease pathology. Moreover, we reveal the profound impact of androgen metabolism in skin and adipose tissue on various aspects of health and disease, particularly in postmenopausal women, where it influences aging, wound healing, obesity, and lipid metabolism.

## 4. Materials and Methods

### 4.1. Chemicals and Reagents

All chemicals, unless otherwise stated, were obtained from Sigma-Aldrich (Taufkirchen, Germany). The following unlabeled and deuterium (d)-labeled reference steroids were purchased as indicated from Sigma-Aldrich, Biomol (Hamburg, Germany), or TCI (Eschborn, Germany): DHEAS (Cay15873, Biomol), DHEA (Cay15728, Biomol), A4 (EPL-DV2, Epichem, Bentley, Australia), T (T0027, TCI), DHT (10300, Sigma-Aldrich), E2 (Cay10006315, Biomol), DHEAS-d_6_ (723266, Sigma-Aldrich), DHEA-d_6_ (ISO-5170, Biomol), A4-^13^C_3_ (ISO-S9044, Biomol), and T-d_3_ (T2655, Sigma-Aldrich). Analytical-grade ultra-pure water (1153332500), methanol (1060352500), and formic acid (5438040100) were from Merck (Darmstadt, Germany). Sep-Pak C18 Plus (360 mg) cartridges (WAT020515) were purchased from Waters Corporation (Milford, MA, USA). The standard compounds were separately dissolved in DMSO at a concentration of 1 mg/mL and stored at −20 °C until further usage. All deuterated standards were diluted with DMSO to their final concentration and mixed to obtain the final IS master mix composition, which was stored at −20 °C until further usage. MCF-7, HDF, and 3T3-L1 adipocyte cells were purchased from the American Type Culture Collection (ATCC, Rockville, MD, USA). The HaCaT cell lines was from AddexBio (San Diego, CA, USA, T0020001).

### 4.2. Cell Culture

The cell culture media and lysates were analyzed using LC-MS/MS, revealing the absence of steroid compounds, including DHEAS, DHEA, A4, T, DHT, and E2, in all samples not treated with DHEAS. MCF-7 and HDF cells were cultured in DMEM (Dulbecco’s modified Eagle medium, Thermo Fisher Scientific, Waltham, MA, USA) supplemented with 10% fetal bovine serum (FBS, Thermo Fisher Scientific), L-glutamine (2 mM, Thermo Fisher Scientific), penicillin (100 U/mL, Thermo Fisher Scientific), and streptomycin (100 µg/mL, Thermo Fisher Scientific) at 37 °C, 5% CO_2_, and 95% humidity. HaCaT cells were maintained in DMEM medium supplemented with 10% FBS, sodium pyruvate (1 mM, Thermo Fisher Scientific), L-glutamine, penicillin/streptomycin at 37 °C, 5% CO_2_, and 95% humidity. Murine 3T3-L1 preadipocytes were cultured as previously reported [57]. Briefly, cultivation occurred in DMEM supplemented with 10% newborn calf serum (NCS; Sigma-Aldrich) at 37 °C and 5% CO_2_. The cells were differentiated into mature adipocytes by culturing them in DMEM/F12/glutamate medium (Lonza, Basel, Switzerland) supplemented with 20 μM 3-isobutylmethylxanthine (Serva, Heidelberg, Germany), 1 μM corticosterone, 100 nM insulin, 200 μM ascorbate, 2 μg/mL apo-transferrin, 5% FBS, 1 μM biotin, 17 μM pantothenate, and 300 μg/mL Pedersen fetuin (MP Biomedicals, Illkirch, France) for 7 days. On the eighth day, cells were switched to a serum-free DMEM/F12/glutamate medium supplemented with 1 µM insulin for 24 h. Finally, mature adipocytes on the ninth day were incubated in serum-free and insulin-free DMEM/F12/glutamate medium for 3–5 h preceding the experiments. At the beginning of the simulation experiments, fresh serum-free medium was supplied to the cells. The cell phenotypes were determined using a bright field under an inverted microscope (Leica DMi1, Wetzlar, Germany) to evaluate the appearance of extensive lipid droplet accumulation. The mature adipocytes after day nine of differentiation were used for stimulation experiments after overnight incubation under serum-free culture conditions. Mature adipocytes were fixed in 4% paraformaldehyde for 15 min. After fixing, the cells were stained with a 4:6 dilution in water of 0.5% Oil Red O (Sigma-Aldrich) in isopropanol for 15 min at 25 °C. The stained cells were then washed twice with distilled water. The lipid droplets were observed in the same setup using a bright field with a 20 x objective. To measure lipid accumulation, the stained cells were dried, and the Oil Red O was extracted in isopropanol. The absorbance was determined at 510 nm using a microplate reader in 96-well plates at the GloMax Multi Detection System (Promega, Tokyo, Japan). Quantitative analysis was performed by comparing the proportional lipid droplet content in preadipocytes and mature adipocytes. The images shown in Figure 6A are representative of the experiments, which were performed independently in triplicate (*n* = 3). All cells were plated on six-well plates at a cell density of 10^6^/well. After 24 h, cells were washed with PBS (137 mM NaCl, 2.7 mM KCl, 1.5 mM KH_2_PO_4_, 7.3 mM Na_2_HPO_4_, pH 7.4, 37 °C), and a serum-free and phenol red-free culture medium was added. The cells were then stimulated by adding 1, 10, or 100 µM DHEAS (in DMSO; final concentration 0.1%). After 24, 48, and 72 h of incubation with DHEAS, cell culture media was collected in microcentrifuge tubes. To measure sex steroids within the remaining cells, cells were washed three times with 1 mL HBSS and 1 mL water was immediately added to each well. Then, the cells were scraped and transferred to clean centrifuge tubes. The cells were lysed using five freeze–thaw cycles. Cell culture media and cell lysates were centrifuged at 12,000× *g* for 10 min. Supernatants were transferred into new tubes and stored at −80 °C until further processing.

### 4.3. Sample Preparation

All plastic vials were rinsed with methanol to rid them of any possible lipophilic contaminants and dried at room temperature. SPE cartridges (Sep-Pak C18 Plus) were successively conditioned with 6 mL of methanol and 6 mL of water prior to use. Frozen cell culture media (1 mL/sample) and cell lysates (1 mL/sample) were thawed at room temperature, mixed with 10 µL of an IS cocktail solution (consisting of DHEAS-d6, DHEA-d6, A4-^13^C_3_, and T-d3), vortexed gently, equilibrated with the IS for 30 min at room temperature, and loaded onto the SPE cartridge. After washing with 3 mL water and drying the cartridges under vacuum for 10 min, analytes were eluted into vials using 2 × 1 mL methanol under 3 psi vacuum and dried under a gentle stream of nitrogen using a sample concentrator (Techne, Cambridge, UK) at room temperature. The dried residues were reconstituted in 100 µL of a mixture containing 55% water (0.1% formic acid) and 45% methanol buffer (0.1% formic acid), resulting in a final IS concentration of 100 ng/mL in the sample. For all samples, the extract was then filtered through 0.2 µm hydrophilic regenerated cellulose syringe filters (Minisart RC4 Syringe Filter 17821K, Sartorius, Goettingen, Germany) and transferred to 2 mL vials with 0.1 mL clear glass micro-inserts (961-10020-20, Shimadzu, Kyoto, Japan) for analysis.

### 4.4. Analysis Using UHPLC-MRM/MS

Sex steroid profiling in cell culture media and cell lysates was performed by reverse-phase UHPLC multiple-reaction monitoring–mass spectrometry (UHPLC-MRM/MS) with negative ion mode for DHEAS and positive ion mode for DHEA, A4, T, DHT, and E2 using Nexera XS inert UHPLC (Shimadzu, Kyoto, Japan) coupled with an API 4000 triple quadrupole mass spectrometer from Applied Biosystems (AB Sciex, Framingham, MA, USA). Chromatographic separation was performed using a Hypersil GOLD aQ C18-LC analytical column (50 mm × 2.1 mm × 3.0 µm) coupled to a Hypersil GOLD aQ C18-LC guard column (10 mm × 2.1 mm × 3.0 µm) with Uniguard direct-connection guard cartridge holders (2.1 mm × 3.0 µm), all from Thermo Fisher Scientific (25303-052130, 25303-012101, and 852-00, respectively). The flow rate was set to 0.50 mL/min and was composed of solvent A (water containing 0.1% formic acid), and solvent B (methanol containing 0.1% formic acid). The gradient used was as follows: T0 min = 45% solvent B; T5–6 min = 95% solvent B; T6–10 min = 45% solvent B. The autosampler was kept at 4 °C, and the column temperature was maintained at 50 °C. For each sample, 10 µL was injected for UHPLC/MRM-MS. The mass spectrometer equipped with an electrospray source in the ESI negative (only for DHEAS and DHEAS-d_6_) or positive polarity mode was configured for MRM to monitor the transitions 367.2 > 96.9 (DHEAS), 289.1 > 271.3, (DHEA), 287.2 > 97.1 (A4), 289.1 > 97.0 (T), 291.1 > 255.3 (DHT), 255.2 > 159.0 (E2), 373.2 > 97.7 (DHEAS-d_6_), 295.1 > 277.1 (DHEA-d_6_), 290.2 > 100.1 (A4-^13^C_3_), and 292.1 > 97.0 (T-d_3_). The electrospray ionization (ESI)–MS/MS conditions included a turbo ion spray voltage of −4500 V (for DHEAS and DHEAS-d6) or 4500 V; a turbo ion spray temperature of 400 °C; ion source gas GS1 and GS2 flows of 20 L/mL; a curtain gas (CUR) flow of 30 L/mL; and a collision gas (CAD) flow of 10 L/min. The MRM transitions in negative and positive ion mode, along with all optimized MS parameters for each analyte, and the IS, including dwell time, declustering potential (DP), entrance potential (EP), collision energy (CE), and collision cell exit potential (CXP), as well as the full scan spectra are shown in Table 1 and Supplementary Figure S1, respectively. Data acquisition and analysis were performed using Analyste 1.6 software (AB Sciex).

### 4.5. Method Validation—Linearity

The validation was performed according to the best industry standards, ICH Q2(R1) [67]. Validation analyses were performed using spiked cell culture medium and cell lysate matrices to ensure precise results. Each calibration curve was accompanied by the processing of cell culture media blanks and cell lysate blanks, which consistently exhibited undetectable levels of steroid hormones. The stock solutions were stored at −20 °C. Standard curve calibrators were prepared for each day of analysis. To evaluate linearity, seven-point calibration plots were prepared for analytes in triplicate at concentrations between 0.5 and 500 ng/mL in different matrices, including cell culture media and cell lysate. To each sample, a cocktail containing the IS at a fixed concentration of 100 ng/mL was added. The ratios of peak areas for the analyte and its corresponding IS were plotted against their respective concentrations. To ensure greater accuracy and precision, a regression with 1/x weighting was selected. The LOD and LLOQ were defined as signal-to-noise ratios of 3 and 10, respectively.

### 4.6. Method Validation—Intra-/Inter-Assay Accuracy and Precision

The precision and accuracy of the assay were evaluated using four different concentrations (1, 3, 75, and 150 ng/mL) in both media and cell lysate samples in triplicate with a sample size of *n* = 6. The following criteria were used to validate precision and intra-assay accuracy: (i) the coefficient of variation (CV) for each concentration level should not exceed 15% for the LLOQ and 20% for quality control (QC) samples; (ii) the mean value of the samples at each concentration level should fall within 85–115% of the actual value of LLOQ and within 80–120% for QC samples.

### 4.7. Method Validation—Recovery and Matrix Effect

To evaluate the recovery of SPE, we compared triplicate QC samples from both cell media and cell lysates, which underwent SPE as described in the sample preparation, with samples of the same concentration obtained by diluting standard compounds only into methanol as the matrix. The calculation involves dividing the mean response of the extracted sample by the mean response of the unextracted sample (spiked blank media or lysate extract sample solution) at the corresponding concentration levels (low-concentration quality control, LQC; medium-concentration quality control, MQC; high-concentration quality control, HQC). For matrix effect experiments, the ratio of spiked standard solutions to the extracted sample residues was used.

### 4.8. Method Validation—Stability

To evaluate stability, we subjected QC media and cell lysate samples (LQC and HQC) to various incubation times ranging from 6 to 56 h at room temperature, +4 °C in the autosampler, and –20 °C in the freezer, as well as four freeze/thaw cycles. Steroid concentrations were then measured and compared with freshly prepared samples.

### 4.9. Statistical Analysis

Graphs and calculations were generated using GraphPad Prism software 6.07 (GraphPad Software, La Jolla, CA, USA). One-way ANOVA was used to compare the means of continuous variables for the time- and concentration-dependent metabolism of DHEAS in cells. Student’s *t*-test was used to compare the means of continuous variables for the lipid accumulation assay in 3T3-L1 cells (Figure 6A). In addition, Student’s *t*-test was used in cases where steroid concentrations were detected at only two time-points (Figure 4). In this case, it cannot be differentiated if the steroid concentrations were truly zero or just under the LLOQ. All data were expressed as mean ± standard deviation (SD). A level of *p* < 0.05 was considered statistically significant.

## Figures and Tables

**Table 1:** Multiple reaction monitoring (MRM) transitions and setting parameters for sex steroid hormones and the stable-isotope-labeled ISs.

Analyte	Polarity (ESI)	Precursor	Parent Ions—Q1 (*m*/*z*)	Daughter Ion—Q3 (*m*/*z*)	RT (min)	Dwell Time (msec)	DP (V)	EP (V)	CE (V)	CXP (V)
**DHEAS**	Negative	[M − H]^−^	367.2	96.9	1.85	150	−115	−10	−56	−15
**DHEA**	Positive	[M − H_2_O + H]^+^	289.1	271.3	3.91	150	66	10	11	18
**A4**	Positive	[M − H_2_O + H]^+^	287.2	97.1	3.21	150	101	10	33	18
**T**	Positive	[M + H]^+^	289.1	97.0	3.65	150	121	10	35	16
**DHT**	Positive	[M + H]^+^	291.1	255.3	4.28	150	71	10	21	16
**E2**	Positive	[M − H_2_O + H]^+^	255.2	159.0	3.42	150	71	10	25	8
DHEAS-d_6_ *	Negative	[M − H]^−^	373.2	97.7	1.82	150	−140	−10	−64	−1
DHEA-d_6_ *	Positive	[M + H]^+^	295.1	277.1	3.87	150	61	10	13	18
A4-^13^C_3_ *	Positive	[M + H]^+^	290.2	100.1	3.20	150	101	10	33	16
T-d_3_ *	Positive	[M + H]^+^	292.1	97.0	3.61	150	101	10	37	16

The stable-isotope-labeled ISs are flagged with asterisks (*). All compounds were analyzed relative to the ISs. ESI, electrospray ionization; RT, retention time; dwell time, the time spent acquiring a specific MRM transition during each cycle; DP, declustering potential; EP, entrance potential; CE, collision energy; CXP, collision cell exit potential; V, volts; *m*/*z*, mass-to-charge ratio.

**Table 2:** Calibration data for steroid hormone analytics.

	Compound	Calibration Range (ng/mL)	Linearity (R^2^)	LOD * (ng/mL)	LLOQ * (ng/mL)
Media	DHEAS	1–500	0.998	0.328	0.994
	DHEA	5–500	0.999	1.822	5.521
	A4	0.5–500	0.998	0.177	0.538
	T	2.5–500	0.997	0.783	2.372
	DHT	2.5–500	0.998	0.726	2.201
	E2	5–500	0.997	1.683	5.100
Cell lysates	DHEAS	1–500	0.998	0.316	0.957
	DHEA	5–500	0.999	1.630	4.938
	A4	0.5–500	0.997	0.159	0.482
	T	2.5–500	0.998	0.787	2.385
	DHT	2.5–500	0.999	0.850	2.575
	E_2_	5–500	0.997	1.669	5.059

* LOD, limit of detection; LLOQ, lower limit of quantification.

## Data Availability

The original contributions presented in this study are included in the article, and further inquiries can be directed to the corresponding authors.

## Supplementary Materials

The following supporting information can be downloaded at: https://www.mdpi.com/article/10.3390/ijms26031188/s1.

## Abbreviations

The following abbreviations are used in this manuscript:

AR	androgen receptor
A4	androstenedione
CE	collision energy
CXP	collision cell exit potential
EP	entrance potential
ER	estrogen receptor
ESI	electrospray ionization
E2	estradiol
DHEA	dehydroepiandrosterone
DHEAS	dehydroepiandrosterone sulfate
DHT	dihydrotestosterone
DP	declustering potential
HDF	human dermal fibroblasts
IS	internal standard
LOD	limit of detection
LLOQ	lower limit of quantification
m/z	mass-to-charge ratio
MRM	multiple-reaction monitoring
QC	quality control
LQC	low-concentration quality control
MQC	medium-concentration quality control
HQC	high-concentration quality control
STS	steroid sulfatase
RT	retention time
V	volts
3β-HSD	3β-hydroxysteroid dehydrogenase
17β-HSD	17β-hydroxysteroid dehydrogenase
