# Supplementary material for: Intracrine Formation of Steroid Hormones in Breast Cancer, Epidermal Keratinocyte, Dermal Fibroblast, and Adipocyte Cell Lines Measured by LC-MS/MS
Source: Int J Mol Sci. 2025 Jan 30;26(3):1188. doi: 10.3390/ijms26031188 (PMC11818555; doi:10.3390/ijms26031188)
Supplement: Supplementary file 1 [file ijms-26-01188-s001.zip › ijms-3417820 Supplementary Files.pdf]

Intracrine formation of steroid hormones in breast cancer, epidermal keratinocyte, dermal fibroblast, and adipocyte cell lines measured by LC-MS/MS

Emre Karakus[1,*], Andreas Schmid[2], Andreas Schäffler[2], Stefan A. Wudy[3], Joachim Geyer[1]

[1] Institute of Pharmacology and Toxicology, Faculty of Veterinary Medicine, Biomedical Research Center Seltersberg (BFS), Justus Liebig University, 35392 Giessen, Germany; Emre.Karakus@vetmed.uni-giessen.de, Joachim.M.Geyer@vetmed.uni-giessen.de

[2] Department of Internal Medicine III, Giessen University Hospital, Justus Liebig University, 35392 Giessen, Germany; Andreas.Schmid@innere.med.uni-giessen.de, Andreas.Schaeffler@innere.med.uni-giessen.de

[3] Steroid Research & Mass Spectrometry Unit, Pediatric Endocrinology and Diabetology, Giessen University Children's Hospital, Justus Liebig University, 35392 Giessen, Germany; Stefan.Wudy@paediat.med.uni-giessen.de

* Correspondence: Emre.Karakus@vetmed.uni-giessen.de

Supplementary Materials

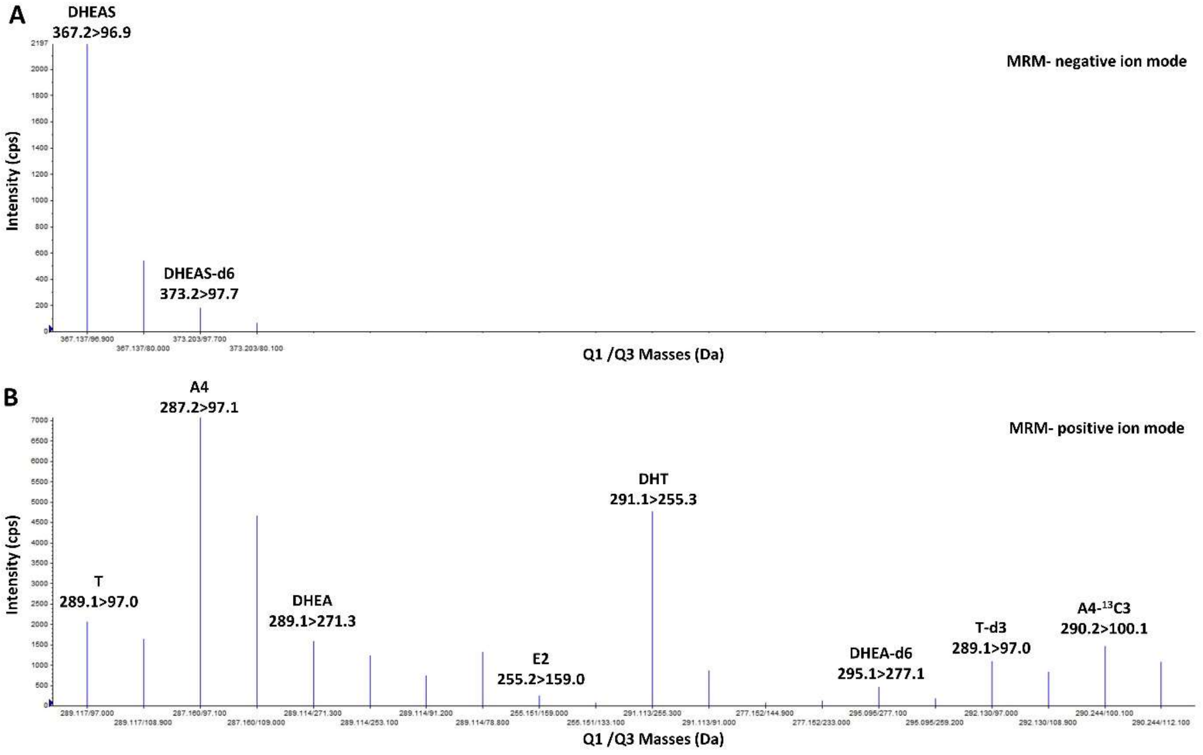

Supplementary Figure S1. Representative negative- (**A**) and positive (**B**) - ESI mass spectra at an individual concentration of 500 ng/mL.

Supplementary Table S1. Accuracy and precision data for steroids from the pre-study validation in media.

	**Nominal value**	**LLOQ**			**LQC**			**MQC**			**HQC**		
	**Batch identification**	**Val.1**	**Val.2**	**Val.3**	**Val.1**	**Val.2**	**Val.3**	**Val.1**	**Val.2**	**Val.3**	**Val.1**	**Val.2**	**Val.3**
**DHEAS**	Intra-assay precision (CV %)	9.2	6.2	1.0	8.2	2.2	7.9	2.6	2.0	3.5	3.0	4.1	3.4
	Intra-assay accuracy (%)	106.8	99.4	105.6	102.0	101.8	97.1	99.3	98.9	100.8	98.4	102.0	99.0
	Inter-assay precision (CV %)	3.1			2.3			0.8			1.6		
	Inter-assay accuracy (%)	103.9			100.3			99.7			99.8		
**DHEA**	Intra-assay precision (CV %)	5.5	3.5	4.6	4.2	3.4	5.2	2.8	4.1	4.7	4.3	4.4	3.2
	Intra-assay accuracy (%)	99.3	109.3	114.0	101.5	105.1	102.3	101.7	102.2	102.9	105.9	105.2	102.1
	Inter-assay precision (CV %)	5.7			1.5			0.5			1.6		
	Inter-assay accuracy (%)	107.5			103.0			102.2			104.4		
**A4**	Intra-assay precision (CV %)	4.4	10.8	9.1	4.1	3.5	7.5	2.3	2.0	2.6	4.4	1.5	3.1
	Intra-assay accuracy (%)	99.4	99.5	100.9	96.6	98.9	102.1	101.5	99.1	102.5	104.5	106.1	98.3
	Inter-assay precision (CV %)	0.7			2.3			1.4			3.3		
	Inter-assay accuracy (%)	100.0			99.2			101.0			103.0		
**T**	Intra-assay precision (CV %)	4.4	6.3	6.7	2.6	3.1	2.9	2.7	2.8	1.7	6.0	6.0	3.8
	Intra-assay accuracy (%)	98.9	105.4	92.3	101.0	100.1	98.8	101.2	100.2	102.9	103.2	103.0	100.8
	Inter-assay precision (CV %)	5.4			0.9			1.1			1.1		
	Inter-assay accuracy (%)	98.9			100.0			101.4			102.3		
**DHT**	Intra-assay precision (CV %)	9.4	6.4	6.3	2.6	6.3	1.3	1.9	7.6	3.0	3.0	3.7	5.4
	Intra-assay accuracy (%)	101.8	106.3	98.2	100.2	102.5	100.2	100.0	91.3	100.9	104.0	95.6	103.8
	Inter-assay precision (CV %)	3.2			1.1			4.4			3.9		
	Inter-assay accuracy (%)	102.1			101.0			97.4			101.1		
**E2**	Intra-assay precision (CV %)	4.8	9.8	4.3	7.4	3.8	6.1	2.4	2.2	2.6	2.7	2.5	4.7
	Intra-assay accuracy (%)	110.0	105.5	104.1	103.6	101.0	107.3	101.5	100.6	101.1	100.6	104.7	101.4
	Inter-assay precision (CV %)	2.4			2.5			0.4			1.7		
	Inter-assay accuracy (%)	106.5			104.0			101.1			102.2		

Val, validation; CV % = [(SD/M) x 100]; Accuracy % = [(E - T)/T] x 100; CV, coefficient of variation; M, mean; SD, standard deviation of M; E, experimentally determined concentration; T, theoretical concentration; LLOQ, limit of quantitation (DHEAS, 1 ng/ml; DHEA and E2, 5 ng/ml;A4, 0.5 ng/ml; T and DHT, 2.5 ng/ml); LQC, low concentration quality control (DHEAS, 3 ng/ml; DHEA and E2, 15 ng/ml; A4, 1.5 ng/ml; T and DHT, 7.5 ng/ml); MQC, medium concentration quality control (DHEAS, DHEA, A4, T, DHT and E2, 75 ng/ml); HQC, high concentration quality control (DHEAS, DHEA, A4, T, DHT and E2, 150 ng/ml).

Supplementary Table S2. Accuracy and precision data for steroids from the pre-study validation in cell lysates.

	Nominal value	LLOQ			LQC			MQC			HQC		
	**Batch identification**	**Val.1**	**Val.2**	**Val.3**	**Val.1**	**Val.2**	**Val.3**	**Val.1**	**Val.2**	**Val.3**	**Val.1**	**Val.2**	**Val.3**
**DHEAS**	Intra-assay precision (CV %)	6.1	3.7	7.3	2.2	3.2	4.2	0.5	1.4	3.2	0.3	0.8	1.9
	Intra-assay accuracy (%)	99.6	102.9	107.1	101.8	94.1	110.6	100.2	99.8	101.6	100.7	101.3	103.4
	Inter-assay precision (CV %)	3.0			6.6			0.8			1.2		
	Inter-assay accuracy (%)	103.2			102.2			100.5			101.8		
**DHEA**	Intra-assay precision (CV %)	3.1	1.4	8.7	2.1	1.6	4.2	0.4	1.6	2.2	0.5	1.5	3.1
	Intra-assay accuracy (%)	103.6	101.5	97.6	101.3	103.4	101.5	100.4	101.1	100.5	100.5	101.0	101.9
	Inter-assay precision (CV %)	2.4			0.9			0.3			0.6		
	Inter-assay accuracy (%)	100.9			102.1			100.7			101.1		
**A4**	Intra-assay precision (CV %)	1.4	1.0	6.6	0.2	2.5	2.9	0.6	5.9	1.8	0.5	2.5	5.0
	Intra-assay accuracy (%)	102.1	95.0	105.0	100.6	95.8	102.5	100.3	101.1	102.2	100.5	101.3	101.4
	Inter-assay precision (CV %)	4.2			2.8			0.8			0.4		
	Inter-assay accuracy (%)	100.7			99.6			101.2			101.1		
**T**	Intra-assay precision (CV %)	2.2	6.7	3.5	2.1	2.5	3.5	3.3	1.7	1.5	3.4	2.2	4.0
	Intra-assay accuracy (%)	100.6	99.6	102.6	100.3	101.2	100.6	101.0	103.6	102.1	104.7	102.0	101.1
	Inter-assay precision (CV %)	1.2			0.4			1.0			1.5		
	Inter-assay accuracy (%)	100.9			100.7			102.2			102.6		
**DHT**	Intra-assay precision (CV %)	1.9	2.9	4.9	0.8	2.3	2.9	1.3	1.6	2.5	2.3	2.0	1.5
	Intra-assay accuracy (%)	101.7	101.0	106.0	100.2	100.6	101.5	101.1	102.9	101.5	101.6	103.6	101.9
	Inter-assay precision (CV %)	2.1			0.5			0.8			0.9		
	Inter-assay accuracy (%)	102.9			100.8			101.9			102.4		
**E2**	Intra-assay precision (CV %)	2.5	3.7	4.7	1.5	4.0	4.2	1.2	2.2	3.2	3.4	2.4	3.4
	Intra-assay accuracy (%)	99.0	96.9	98.5	99.4	99.4	103.8	100.7	102.2	100.3	99.9	103.1	103.0
	Inter-assay precision (CV %)	0.9			2.1			0.8			1.5		
	Inter-assay accuracy (%)	98.1			100.9			101.1			102.0		

Val, validation; CV % = [(SD/M) x 100]; Accuracy % = [(E - T)/T] x 100; CV, coefficient of variation; M, mean; SD, standard deviation of M; E, experimentally determined concentration; T, theoretical concentration; LLOQ, limit of quantitation (DHEAS, 1 ng/ml; DHEA and E2, 5 ng/ml; A4, 0.5 ng/ml; T and DHT, 2.5 ng/ml); LQC, low concentration quality control (DHEAS, 3 ng/ml; DHEA and E2, 15 ng/ml; A4, 1.5 ng/ml; T and DHT, 7.5 ng/ml); MQC, medium concentration quality control (DHEAS, DHEA, A4, T, DHT and E2, 75 ng/ml); HQC, high concentration quality control (DHEAS, DHEA, A4, T, DHT and E2, 150 ng/ml).

Supplementary Table S3. Recovery and matrix effect for steroids in media and cell lysate.

Matrix	Analyte		LQC			MQC			HQC		
			Standard solution	Spiked blank media or lysate extract sample solution	Extracted sample solution	Standard solution	Spiked blank media or lysate extract sample solution	Extracted sample solution	Standard solution	Spiked blank media or lysate extract sample solution	Extracted sample solution
Media	DHEAS	Precision (CV%)	8.3	1.3	13.3	2.7	13.4	9.0	10.8	13.0	12.7
		Recovery (%)	120.4			104.6			104.2		
		Matrix Effect (%)	111.6			110.1			112.3		
	DHEA	Precision (CV%)	8.7	7.2	6.8	13.4	9.1	3.5	8.4	3.6	12.3
		Recovery (%)	101.5			90.5			89.0		
		Matrix Effect (%)	104.3			99.9			90.2		
	A4	Precision (CV%)	11.0	8.1	9.4	10.5	9.9	7.6	8.5	11.3	4.7
		Recovery (%)	93.5			95.0			108.8		
		Matrix Effect (%)	108.4			89.2			104.6		
	T	Precision (CV%)	7.1	8.2	1.2	7.3	0.4	1.3	9.5	13.0	9.0
		Recovery (%)	101.8			115.2			101.2		
		Matrix Effect (%)	109.3			103.4			92.2		
	DHT	Precision (CV%)	13.2	8.9	7.5	2.3	9.3	7.6	9.3	12.7	9.6
		Recovery (%)	100.3			99.5			99.5		
		Matrix Effect (%)	113.6			87.7			104.7		
	E2	Precision (CV%)	10.9	13.3	5.4	3.9	12.6	5.1	10.3	13.7	8.2
		Recovery (%)	97.7			102.0			98.2		
		Matrix Effect (%)	113.8			90.1			90.8		
Cell lysate	DHEAS	Precision (CV%)	14.4	11.8	5.2	11.9	5.1	14.1	5.4	6.0	13.3
		Recovery (%)	92.3			105.0			112.0		
		Matrix Effect (%)	108.7			92.7			95.5		
	DHEA	Precision (CV%)	13.7	5.5	10.9	12.0	15.9	11.3	3.0	6.1	8.7
		Recovery (%)	110.0			88.4			91.2		
		Matrix Effect (%)	83.2			93.9			86.3		
	A4	Precision (CV%)	7.8	8.7	11.3	9.3	4.0	14.3	14.5	6.5	9.3
		Recovery (%)	104.2			107.0			114.1		
		Matrix Effect (%)	110.2			91.3			96.5		
	T	Precision (CV%)	11.0	10.2	11.0	5.2	2.6	10.6	6.0	11.0	11.0
		Recovery (%)	96.7			95.8			113.5		
		Matrix Effect (%)	96.5			103.7			95.9		
	DHT	Precision (CV%)	8.3	14.6	14.7	7.5	9.9	10.5	10.7	10.3	3.5
		Recovery (%)	94.9			101.4			107.1		
		Matrix Effect (%)	94.9			108.8			105.5		
	E2	Precision (CV%)	9.1	13.0	14.0	10.4	4.9	14.2	13.9	8.8	7.7
		Recovery (%)	95.6			115.2			117.0		
		Matrix Effect (%)	97.1			104.4			94.9		

CV %, coefficient of variation = [(SD/M) x 100]; recovery (%) = [(M of extracted sample solution /M of spiked blank media or lysate extract sample solution) x 100]; matrix Effect (%) = [(M of extracted sample solution /M of standard solution) x 100]; M, mean; SD, standard deviation of M; LQC, low concentration quality control (DHEAS, 3 ng/ml; DHEA and E2, 15 ng/ml; A4, 1.5 ng/ml; T and DHT, 7.5 ng/ml); MQC, medium concentration quality control (DHEAS, DHEA, A4, T, DHT and E2, 75 ng/ml); HQC, high concentration quality control (DHEAS, DHEA, A4, T, DHT and E2, 150 ng/ml).

Supplementary Table S4. Stability tests for steroids in media and cell lysates.

Matrix	Analyte		Freeze/thaw		RT						+ 4°C stability						- 20°C stability					
			LQC	HQC	LQC			HQC			LQC			HQC			LQC			HQC		
					6 h	24 h	56 h	6 h	24 h	56 h	6 h	24 h	56 h	6 h	24 h	56 h	6 h	24 h	56 h	6 h	24 h	56 h
Media	DHEAS	Precision (CV%)	3.3	3.5	4.2	1.4	3.2	3.5	2.8	2.2	1.4	2.1	2.8	3.7	3.5	1.3	2.7	11.8	7.4	2.3	4.9	3.2
		Accuracy (%)	105.3	99.9	102.1	100.7	98.3	97.8	104.1	107.5	103.3	109.1	100.0	100.2	99.2	99.7	102.5	111.0	102.8	99.9	102.4	99.8
	DHEA	Precision (CV%)	2.8	3.0	3.2	0.3	1.6	1.8	3.1	3.4	6.5	3.4	1.0	3.8	1.4	4.6	2.9	3.4	0.3	4.0	1.9	1.0
		Accuracy (%)	104.6	103.0	99.2	90.7	93.9	91.2	93.0	92.8	101.7	101.4	99.2	105.4	99.7	104.0	100.0	99.5	102.3	104.1	108.7	102.8
	A4	Precision (CV%)	2.3	4.8	1.6	0.9	0.6	0.3	1.2	0.4	3.1	2.2	1.5	2.0	2.9	4.0	7.8	8.8	3.7	2.6	5.0	2.4
		Accuracy (%)	104.3	101.8	91.2	87.8	89.0	91.4	85.6	90.6	102.8	96.9	100.9	104.8	101.0	107.3	98.6	98.0	102.0	103.1	100.3	104.4
	T	Precision (CV%)	0.8	3.8	3.4	2.1	2.0	3.2	3.1	1.7	0.8	0.4	3.2	2.3	4.1	2.8	0.5	2.6	4.5	3.6	3.3	2.6
		Accuracy (%)	96.5	101.6	99.5	94.2	103.2	101.6	101.8	89.7	104.3	96.3	96.5	98.0	91.5	102.5	103.7	100.0	101.7	103.9	100.5	99.4
	DHT	Precision (CV%)	2.4	3.4	3.8	2.5	3.8	1.4	6.2	1.7	1.2	0.7	2.5	4.6	5.4	2.8	4.1	1.2	1.3	3.3	6.2	6.4
		Accuracy (%)	99.8	100.9	101.8	98.8	100.4	106.7	103.0	88.7	96.6	96.9	99.3	106.0	102.9	98.1	100.9	99.9	100.3	101.1	107.6	104.1
	E2	Precision (CV%)	3.0	1.0	5.9	5.6	4.0	4.6	3.3	0.6	4.5	3.9	6.8	5.8	3.8	3.6	1.7	2.8	3.8	1.2	5.5	2.1
		Accuracy (%)	102.5	104.2	103.8	104.2	104.3	107.4	90.7	90.2	102.8	106.1	99.5	101.5	100.0	106.3	96.8	102.6	102.5	101.7	102.9	109.8
Cell lysates	DHEAS	Precision (CV%)	7.6	2.4	8.5	5.9	2.1	2.5	3.1	5.3	3.1	2.8	1.3	3.3	3.3	1.7	7.9	3.3	2.4	4.0	3.3	2.6
		Accuracy (%)	104.9	103.1	100.6	104.2	104.3	99.7	100.3	102.6	99.1	100.4	93.1	103.9	100.5	104.1	102.2	108.2	93.4	102.9	105.6	97.0
	DHEA	Precision (CV%)	2.7	2.0	0.9	6.4	7.5	5.0	5.8	1.7	3.3	5.3	20.4	2.6	2.3	2.2	2.9	2.0	3.3	5.5	3.8	3.6
		Accuracy (%)	103.1	103.9	108.3	102.8	104.9	103.2	103.7	114.1	98.6	102.7	92.6	109.7	103.7	99.2	100.1	104.8	105.9	100.3	100.5	106.9
	A4	Precision (CV%)	3.7	1.9	1.3	2.7	2.4	2.0	2.6	2.8	0.3	1.7	2.8	4.2	1.5	3.5	3.4	2.3	2.5	5.5	6.4	2.8
		Accuracy (%)	103.8	102.4	113.1	91.2	100.1	104.7	91.8	87.0	99.7	102.9	105.8	101.1	104.7	102.3	102.1	99.8	103.3	105.0	104.5	104.7
	T	Precision (CV%)	2.4	1.2	3.0	1.7	1.8	2.4	1.6	1.5	2.0	2.3	2.2	1.5	1.3	3.1	4.2	4.0	1.4	3.0	1.3	4.1
		Accuracy (%)	104.0	105.3	98.3	87.5	87.4	96.4	87.9	90.1	104.0	97.0	100.3	99.7	105.4	102.3	93.4	99.7	97.4	102.0	107.1	102.9
	DHT	Precision (CV%)	3.4	2.9	1.3	1.1	3.7	3.1	1.8	2.3	4.0	3.9	0.5	1.3	3.3	6.6	2.8	1.3	4.4	2.1	4.6	1.6
		Accuracy (%)	97.7	102.9	88.2	86.1	87.8	88.6	87.5	88.6	99.9	101.8	98.7	97.4	98.7	102.3	98.7	100.6	99.2	109.6	98.8	110.3
	E2	Precision (CV%)	2.8	2.8	2.2	0.2	1.3	0.8	2.9	2.3	6.6	1.6	5.5	3.4	1.0	1.9	5.8	4.9	4.4	4.2	2.1	4.8
		Accuracy (%)	103.0	103.7	90.1	88.3	89.0	87.6	90.9	90.6	103.7	99.3	101.3	100.8	100.2	109.3	104.3	102.9	99.2	99.8	106.7	102.9

CV %, coefficient of variation = [(SD/M) 100]; M, mean; SD, standard deviation of M; LQC, low concentration quality control (DHEAS, 3 ng/ml; DHEA and E2, 15 ng/ml; A4, 1.5 ng/ml; T and DHT, 7.5 ng/ml); HQC, high concentration quality control (DHEAS, DHEA, A4, T, DHT, and E2 150 ng/ml); freeze/thaw was 4 cycles.
